# Prefoveal floaters as a differential diagnosis to optic neuritis: “mouches dormantes”

**DOI:** 10.1007/s13760-017-0810-y

**Published:** 2017-06-17

**Authors:** Marloes C. Burggraaff, Willemine A. E. J. de Vries-Knoppert, Axel Petzold

**Affiliations:** 1 Neuro-Ophthalmology Expertise Centre, VU University Medical Center, Amsterdam, The Netherlands; 2 Department of Neuro-Ophthalmology, Moorfields Eye Hospital, City Road, London, UK

**Keywords:** Optical coherence tomography, Floater, Mouche, Endoptic phenomena, Optic neuritis

## Abstract

This case series describes a new optical coherence tomography (OCT) specific observation relevant to the differential diagnosis of patients with suspected optic neuritis. A tiny prefoveal floater, only detectable by OCT, was found responsible for the symptoms in three patients, one of whom had been referred with unilateral delayed visual evoked potentials. This case series suggests that with increased use of OCT in routine clinical care, entoptic phenomena can be demonstrated as a relevant differential diagnosis to optic neuritis. Patients should be explained the benign nature of their symptoms.

## Introduction

Should optical coherence tomography (OCT) be part of the routine clinical assessment is a controversially debated topic in the neuro-ophthalmology community [1]. There are good arguments against routine use of OCT [2, 3], and situations were a good case can be made for it [4, 5]. This series of cases referred to use with suspected optic neuritis, highlights that OCT is also useful to diagnose dormant entopic phenomena, which can be confusing for both patients and physicians.

Entoptic phenomena due to floaters, synonymous mouches volantes or vitreous opacities are common. They can result in shadowing artefacts on the retina [6, 7]. Patients who present with a visual field defect (VFD) may have a so-called floater scotoma [8]. The differential diagnosis includes retinal and optic nerve pathology [5].

## Case series

Case review of three subsequent patients is seen between November and December 2014 in Amsterdam and London. Macular volume scans were conducted with spectral-domain OCT (Heidelberg Spectralis at both Institutions).

## Case 1

**Fig. 1:**
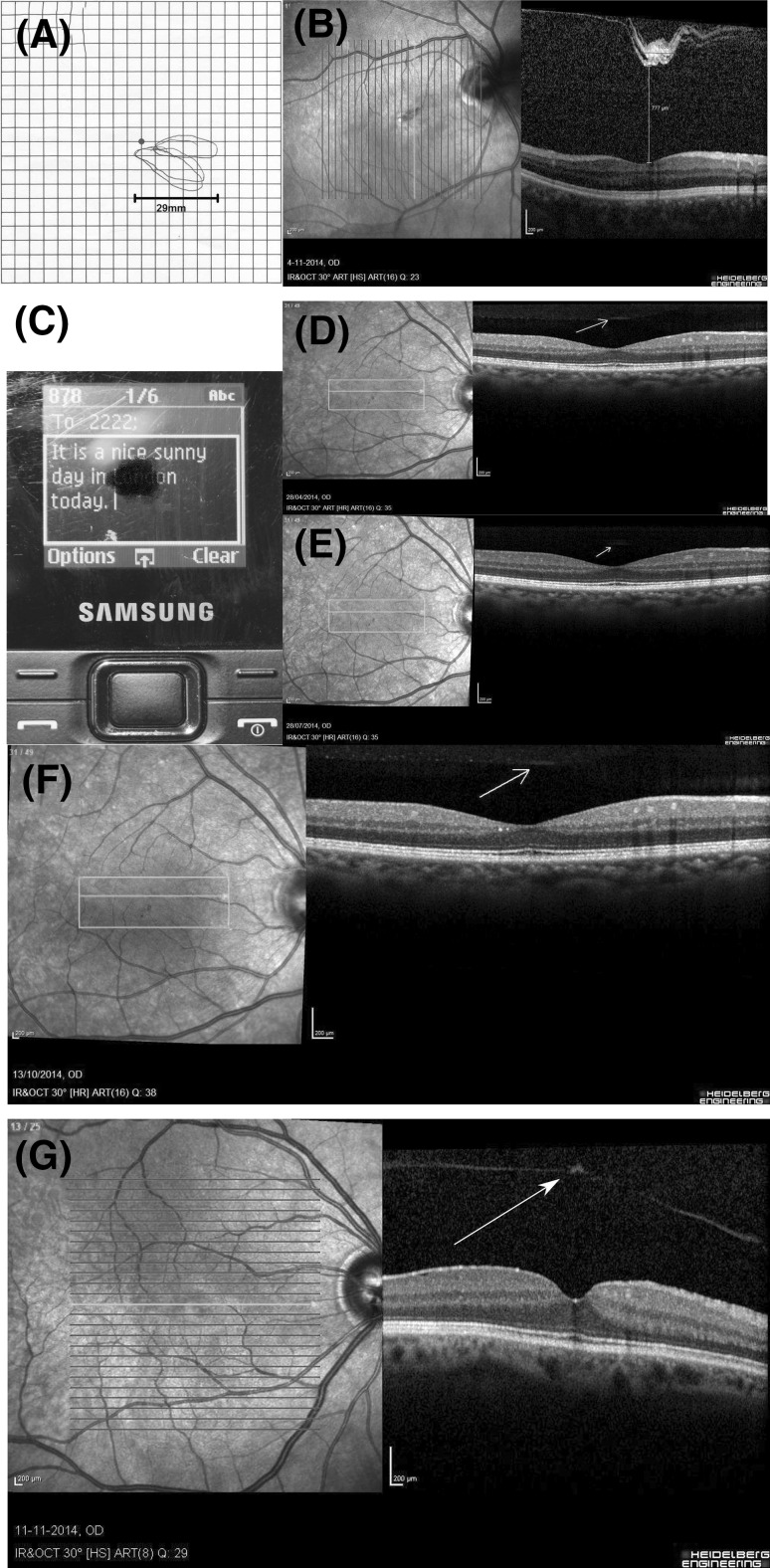
**a** Amsler chart, case 1. A central scotoma, due to a prefoveal floater, extending to 2.2° × 1.3° of visual angle on the Amsler chart held at about 30 cm distance such that one *square* corresponds to 1°. **b** Optical coherence tomography, case 1. A prefoveal floater is seen on the *right* (cSLO). The 25 vertical *green lines* correspond to subsequent OCT B-scans on an approximate 30° field. The distance between the individual *lines* calculates to 1.2°. The floater measures about 3.6° × 1.2° on the cSLO image. This prefoveal floater corresponds to the mirror image shown on the Amsler chart in **a**. **d** Central scotoma in case 2. The small, relative central scotoma makes it difficult to read small print text messages on his smart phone. The text for the city of “London” is obscured whilst focusing on the words “nice sunny”. **e–f** Serial OCT was taken over a 9-month period. One small prefoveal floater remains in exactly the exact same place at three-month follow-up visits. **g** The very small prefoveal floater in case 3 casts a dense shadow in the OCT image, which is not visible on the sCLO image on the *right*, but can be seen on the OCT B-scan on the *left* (colour figure online)

A 39-year-old, myopic (−6 dpt in both eyes) man experienced a central scotoma in his right eye for the past year. The central scotoma considerably interfered with his work on a computer screen. He described a grey/black spot which he could clearly delineate it on an Amsler chart (see Fig. 1a). The scotoma extended to approximately 2.2° × 1.3° of visual angle and was static centrally, but moved in an about 45° angle to the periphery.

His best-corrected visual acuity (BCVA) was 0.6 on the right and 1.0 on the left. Optic neuritis was suspected elsewhere and visual evoked potentials (VEP) were performed. Because of an asymmetric latency of the VEP (108.6 ms on the right, compared to 101.4 ms on the left), he was referred to us for suspected optic neuritis. The macular volume OCT scan showed vitreous opacifications in front of the fovea (Fig. 1b). The location and shape corresponded to the scotoma seen on the Amsler chart (Fig. 1a). The size of the floater (which was 777 µm from the fovea) on OCT was approximately 1035 µm, whereas the size of the perceived floater on the Amsler chart (at a viewing distance of about 30 cm) was 29 mm, therefore the enlargement perceived by the patient was 28 times.

## Case 2

A 41-year-old IT-consultant had experienced photopsia in his good right eye which were followed by a small greyish smear or patch in his central vision. The relative scotoma did interfere with reading. The left eye was amblyopic. He had also experienced ocular pain and was referred to us with suspected optic neuritis. Whilst the recurrent photopsia and peri-ocular pain could be explained by a visual aura and migraine, the static VFD could not. The VFD was located slightly inferior to the centre in the right eye. Whilst he could see through the smear, he found it difficult to read a text message on his smart phone (Fig. 1c). Revision of his previous three serial macular volume OCT scans reproducibly showed a small prefoveal floater which did only cast a very faint shadow (Fig. 1d–f).

## Case 3

A 67-year-old man reported progressive visual decline and VFD in his right eye. His BCVA was 0.4 on the right and 0.8 on the left. He was referred with a suspected optic neuropathy. On clinical examination there was also significant bilateral cataract. Fundoscopy was normal. The automatic perimetry showed decreased central sensitivity. Colour vision was normal. Brain MRI did show normal signal and size of the optic pathways. After being referred to us, retinal OCT imaging revealed a small prefoveal floater and fibrosis of the internal limiting membrane (Fig. 1g). Note that despite the small size of this floater the optical density is high which cast a shadow that is clearly seen through all retinal layer in the central foveola.

## Discussion

Referrals to a neuro-ophthalmology unit include optic neuritis and optic neuropathies [1, 2, 5]. This case series shows that small prefoveal floaters just dense enough to cast a shadow on the retina were the cause for the patients’ central scotomas.

There were features which understandably lead to a referral diagnosis of optic neuritis or another optic neuropathy. Further investigation either by automatic perimetry and results of the VEP, seemingly supported the referral diagnosis. There are however a large number of reasons why VEPs can be delayed and a prefoveal floater causing reduced visual acuity may be added to the list including poor refraction, cataract, migraine  and many other causes [5].

Floater scotomas may occur in eyes with or without posterior vitreous detachment (PVD), incomplete PVD or vitreous cysts [7, 8]. To this list small prefoveal floaters only detectable by OCT may be added. Prefoveal floaters differ from typical mouches volantes as their location does not permit for the so typical lateral movements clearly described for larger and more distally located floaters. Tentatively one may suggest the term ‘mouches dormantes’ (French ‘dormant’ translates to ‘sleeping’) as they seem to lay sleeping until detection by OCT.

With regard to patient management the most relevant point is to explain the benign nature of a floater. If the vision of a patient is severely effected by a large floater, pars plana vitrectomy can be effective. For the small prefoveal floaters reported here, there is a lack of evidence for both surgical and laser invention. One group performed a Nd:YAG laser posterior hyaloidotomy for a premacular vitreous floater with poor outcome [9]. Therefore we do not recommend any invasive procedures for small prefoveal floaters. Likewise, ocriplasmin which can induce posterior vitreous detachment is currently not advocated for floaters.

We suspect that with the increasing use of handheld visual devices requiring a pristine central visual field (such as smart phones and tablets), more of these ‘mouche dormants’ will be recognised by broader routine clinical use of OCT. For patients this implies that they can be reassured about the benign nature of their symptoms, rather than remaining anxious about potentially sinister neurological pathology in absence of evidence to the contrary.

## References

[1] Costello F, Van Stavern GP (2012). Should optical coherence tomography be used to manage patients with multiple sclerosis?. J Neuroophthalmol.

[2] Hickman SJ (2011). Neuro-ophthalmology. Pract Neurol.

[3] Jenkins TM, Toosy AT (2014). Optical coherence tomography should be part of the routine monitoring of patients with multiple sclerosis: no. Mult Scler.

[4] Saidha S, Calabresi PA (2014). Optical coherence tomography should be part of the routine monitoring of patients with multiple sclerosis: yes. Mult Scler.

[5] Petzold A, Wattjes MP, Costello F, Flores-Rivera J, Fraser CL, Fujihara K, Leavitt J, Marginier R, Paul F, Schippling S, Sindic C, Villoslada P, Weinshenker B, Plant GT (2014). The investigation of acute optic neuritis: a review and proposed protocol. Nat Rev Neurol.

[6] Schippling S, Balk L, Costello F, Albrecht P, Balcer L, Calabresi P, Frederiksen J, Frohman E, Green A, Klistorner A, Outteryck O, Paul F, Plant G, Traber G, Vermersch P, Villoslada P, Wolf S, Petzold A (2015). Quality control for retinal OCT in multiple sclerosis: validation of the OSCAR-IB criteria. Mult Scler.

[7] Kennelly KP, Morgan JP, Keegan DJ, Connell PP (2015). Objective assessment of symptomatic vitreous floaters using optical coherence tomography: a case report. BMC Ophthalmol.

[8] Schwartz SG, Flynn HW, Fisher YL (2013). “Floater scotoma” demonstrated on spectral-domain optical coherence tomography and caused by vitreous opacification. Ophthalmic Surg Lasers Imaging Retina.

[9] Van der Veken A, Van de Velde F, Smeets B, Tassignon MJ (1997). Nd:yAG laser posterior hyaloidotomy for the treatment of a premacular vitreous floater. Bull Soc Belge Ophtalmol.

